# In-vitro modelling of Alzheimer’s disease using cholinergic neurons derived from human neuroblastoma (SH-SY5Y) retinoic acid-induced differentiation

**DOI:** 10.1007/s11033-026-12156-4

**Published:** 2026-06-23

**Authors:** Muhammad-Safuan Zainuddin, Kasthuri Bai Magalingam, Narendra Pamidi, Adzzie Shazleen Azman, Saatheeyavaane Bhuvanendran

**Affiliations:** 1 Jeffrey Cheah School of Medicine and Health Sciences, Monash University Malaysia, Jalan Lagoon Selatan, Bandar Sunway, Petaling Jaya, 47500 Selangor Malaysia; 2 School of Science, Monash University Malaysia, Jalan Lagoon Selatan, Bandar Sunway, Petaling Jaya, 47500 Selangor Malaysia

**Keywords:** Alzheimer’s disease, neuronal differentiation, cell differentiation, cholinergic neurons, retinoic acid, cytotoxicity

## Abstract

**Background:**

Alzheimer’s disease (AD) is characterised by severe degeneration of cholinergic neurons within the basal forebrain complex (FBC) which is a key regulator of cognitive function. Cholinergic loss represents a central pathological hallmark of AD; however, the underlying molecular mechanisms remain incompletely understood. Although various in-vitro models are available, many are limited by species-specific differences, high cost, and technical complexity. Human neuroblastoma (SH-SY5Y) cells can be differentiated into neuron-like cells and represent a practical alternative for AD research. This study aimed to optimise retinoic acid (RA)-based differentiation conditions to enhance cholinergic characteristics in SH-SY5Y cells and evaluate their susceptibility to AD-related stressors as a simplified, cost-effective model for preliminary high-throughput AD studies.

**Methods:**

A structured literature search (2000–2025) was conducted using PubMed and ScienceDirect. After screening based on predefined criteria, 23 relevant studies were analysed for differentiation inducers, serum concentration, duration, neuronal markers, and cholinergic markers. Here, a simplified RA-only protocol was evaluated using 10µM RA with 1% or 3% heat-inactivated foetal bovine serum (1% or 3% HI-FBS) over 3, 5, and 7 days. Neuronal differentiation was assessed by morphological analysis, neurite length measurement, choline acetyltransferase (*ChAT*) and acetylcholinesterase (*AChE*) gene expressions, acetylcholinesterase (AChE) activity. Additionally, model relevance was further evaluated using AD-associated stressors such as streptozotocin (STZ), hydrogen peroxide (H₂O₂), lipopolysaccharide (LPS), and aluminium chloride (AlCl₃).

**Results:**

Although most protocols generated mature neuron-like cells, only ~ 30% reported cholinergic marker expression, with retinoic acid (RA) and brain-derived neurotrophic factor (BDNF) as the most common inducers. This study reports that differentiation with 1% HI-FBS with 10µM RA for 7 days produced pronounced neuronal morphology, significant neurite extension, and extensive branching. These cells demonstrated a cholinergic-like phenotype, with significant upregulation of *ChAT* and *AChE* gene expressions, accompanied by increased AChE enzymatic activity. These neuron-like cells also showed dose-dependent responses to STZ, H₂O₂, and AlCl₃, with time-dependent effects observed for H₂O₂ and AlCl₃. Notably, cells were resistant to LPS-induced cytotoxicity.

**Conclusion:**

These findings support the utility of this RA-differentiated SH-SY5Y for neuronal-like cells for cholinergic-like model (1% HI-FBS, 10µM RA) as a practical and cost-effective platform for high-throughput AD drug screening.

**Graphical abstract:**

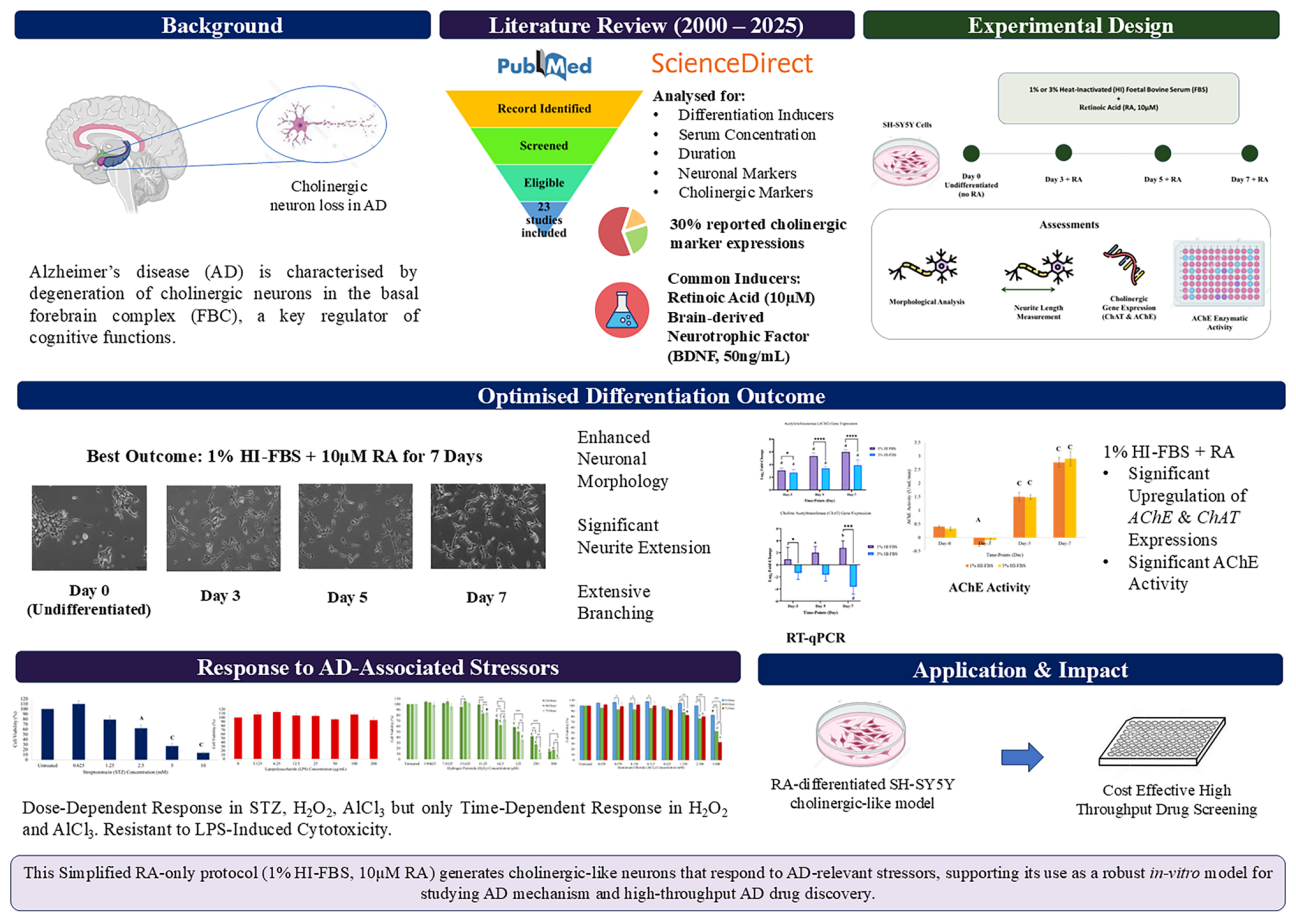

**Supplementary Information:**

The online version contains supplementary material available at 10.1007/s11033-026-12156-4.

## Introduction

Alzheimer’s disease (AD) is a major global health challenge due to its rapidly increasing prevalence and progressive cognitive decline, including impairments in memory, reasoning, and behaviour that culminate in dementia [1–4]. A key pathological feature of AD is the degeneration of cholinergic neurons in the basal forebrain complex, which innervate cognition-associated regions such as the neocortex and hippocampus [5–7]. Despite extensive research, the precise molecular mechanisms underlying AD remain incompletely understood, partly due to the lack of suitable in-vitro models that recapitulate mature human cholinergic neurons.

Conventional neuronal models, including murine-derived cell lines (e.g., B35, Neuro-2 A, PC12, MN9D) and human induced pluripotent stem cell (hiPSC)-derived neurons, present notable limitations such as species-specific differences, high cost, and technical complexity [8–10]. Human neuroblastoma (SH-SY5Y) cells provide a cost-effective and reproducible alternative. This heterogeneous cell line, derived from SK-N-SH, can be differentiated using retinoic acid (RA), with or without neurotrophic factors such as brain-derived neurotrophic factor (BDNF) or nerve growth factor (NGF), to exhibit neuronal phenotypes characterized by neurite outgrowth and increased expression of mature neuronal markers [8, 11–15]. Importantly, SH-SY5Y cells can express cholinergic and other neurotransmitter-related markers following differentiation, making them a practical platform for neurodegenerative research [11, 15–18]. In this study, we evaluated RA-based differentiation protocols to optimize serum concentration and differentiation duration for enhancing cholinergic features and subsequently assessed the susceptibility of these differentiated cells to AD-related stressors as a simplified and cost-effective in-vitro model for preliminary high-throughput AD studies.

## Materials and methods

### Literature search on available differentiation protocols

A literature search was conducted to identify studies describing differentiation protocols for in-vitro SH-SY5Y model. Two electronic databases (PubMed and ScienceDirect) were searched for relevant original research articles published within the past 25 years (January 2000 – December 2025). The search strategy incorporated the following keywords and subject headings: “human neuroblastoma”, “SH-SY5Y”, “Differentiation”, “Differentiated”, “Neuronal”, “Cholinergic”, “Alzheimer’s disease”, and “retinoic acid”. These terms were applied to titles, abstracts, and Medical Subject Headings (MeSH) using appropriate Boolean operators for defined search results. And a series of inclusion and exclusion criteria were used (Table 1).

**Table 1:** Literature search inclusion and exclusion criteria for differentiation protocols of in-vitro AD model

Inclusion	Exclusion
English Language	Non-English Language
Human neuroblastoma (SH-SY5Y)	Non-human neuroblastoma or non-SH-SY5Y
Differentiation Protocol	Non-Differentiation Protocol
In-vitro	In-vivo, clinical studies
Original research articles	Review, systematic review, meta-analysis

### Human neuroblastoma (SH-SYSY) cell culture and maintenance

Human neuroblastoma (SH-SY5Y) cells (CRL-2266) was purchased from the American Type Culture Collection (ATCC). The cells were maintained in complete media (CM) consisting of high-glucose Dulbecco’s Modified Eagle Medium (DMEM) (25mM D-glucose, 4.0mM L-Alanyl-L-Glutamine, 1.0mM Sodium Pyruvate) (HiMedia, India), 10% (V/V) heat-inactivated foetal bovine serum (HI-FBS) (Gibco™) (ThermoFisher Scientific, USA) and 1% (V/V) penicillin-streptomycin (P/S) (Gibco™) (ThermoFisher Scientific, USA). The cells were cultured in T-75 flasks at 37 °C in a humidified atmosphere (5% CO_2_) incubator. The culture medium was replaced every two days with fresh medium and subculture at 70–80% confluence. The confluent cells were detached mechanically using cell scrapper in 1X phosphate buffer saline (1X PBS, pH 7.4) (Sigma-Aldrich, USA) in minimising surface receptor damages. Only attached cells were maintained and floating cells were discarded. The passage number of the cells were maintained and limited between passage 15–20 to avoid phenotypic and morphological drift [9].

### Differentiation protocol and experimental group division

**Fig. 1:**
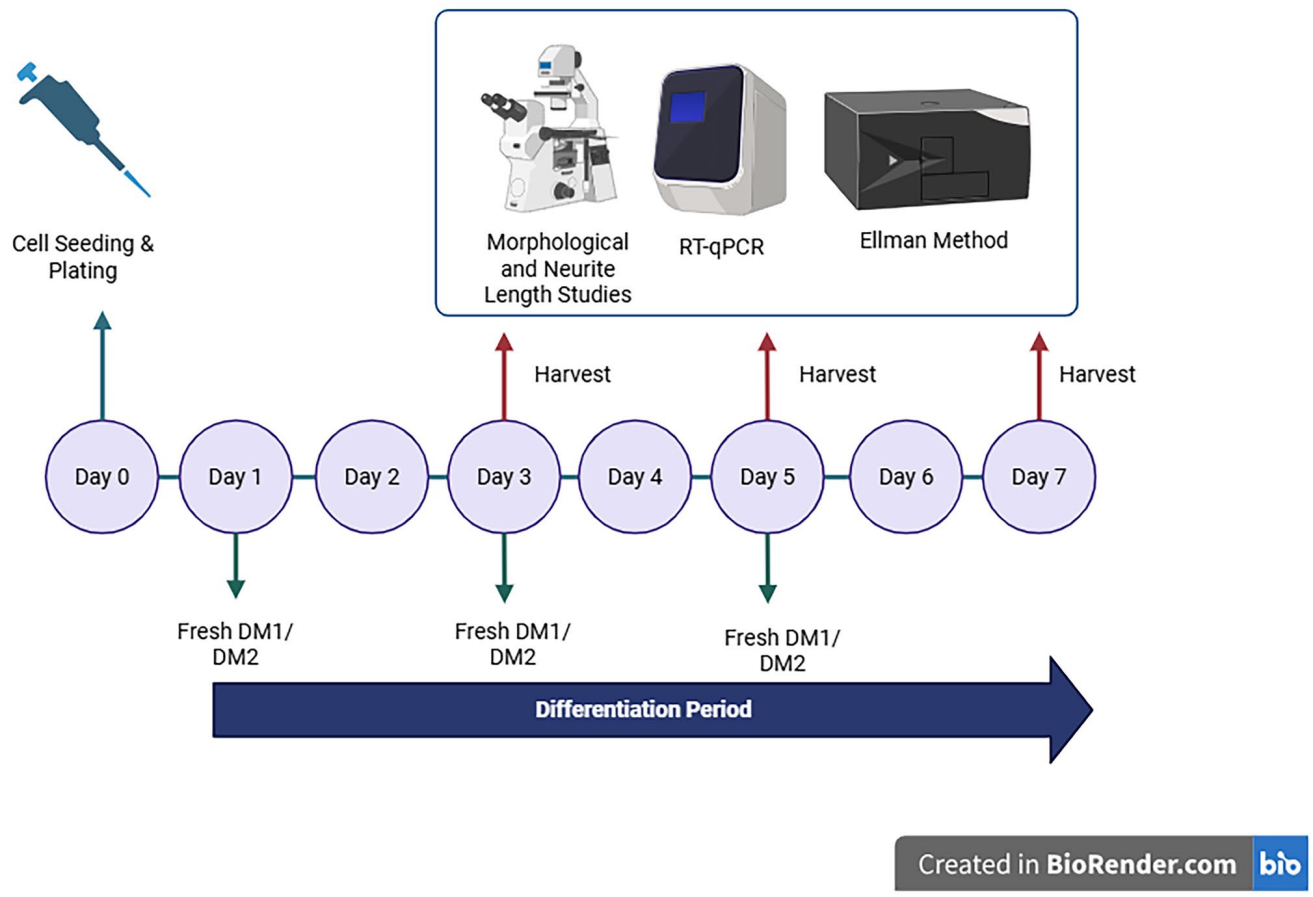
Differentiation protocols. The proliferative human neuroblastoma (SH-SY5Y) cells were seeded (150,000 cells/mL) and cultured in complete media (CM) containing high-glucose DMEM, 10% (V/V) HI-FBS), and 1% (V/V) P/S for 24-hour for complete adhesion. Afterwards, two differentiation media (DM) containing high-glucose DMEM, 1% (V/V) P/S, 10µM RA, and supplemented with either 1% (V/V) HI-FBS (DM1) or 3% (V/V) HI-FBS (DM2). The cells were differentiated at three time-points: Day 3, Day 5, and Day 7. At the indicated days, the cells were harvested and underwent several analyses; (i) morphological, (ii) neurite length, (iii) RT-qPCR, and (iv) Ellman method

To systematically evaluate the effect of serum concentration (1% HI versus 3% HI) across distinct differentiation time points (Day 3, Day 5, and Day 7) on SH-SY5Y differentiation induced by 10µM retinoic acid (RA), addressing variability reported in the literature and aiming to optimize a simplified, reproducible protocol based solely on RA for the induction of cholinergic-like neuronal features. The SH-SY5Y differentiation was carried out by seeding 1.5 × 10^5^ cells/mL onto 96-well plates, T-25 flasks, and T-75 flasks based on experimentation. The cells were seeded in complete media (CM) containing high glucose DMEM, 10% (V/V) HI-FBS, and 1% (V/V) P/S. The cells were left to adhere overnight. Afterwards, the SH-SY5Y cells were subjected to two differentiation medias (DMs) containing high glucose DMEM, 1% (V/V) P/S, 10µM RA, and supplemented with either 1% (V/V) HI-FBS (DM1) or 3% (V/V) HI-FBS (DM2). The cells were left to differentiate for 3, 5, and 7 days at 37 °C in a humidified atmosphere (5% CO_2_). The spent media was replaced with either fresh DM1 or DM2 every 2 days. The differentiated cells were harvested at indicated days and underwent morphological, Ellman method, and gene expression analyses (Fig. 1). The RA was dissolved in analytical grade DMSO (ThermoFisher Scientific, USA) with the final concentration in all treatment groups were 0.1% (V/V) which is below the standard threshold for cell-based assay.

### Morphological study

The SH-SY5Y cells (1.5 × 10^5^ cells/mL) were cultured in T-25 flasks and treated accordingly. The phenotypic characteristics of undifferentiated and differentiated SH-SY5Y at the indicated days were captured using CKX41 inverted microscope (Olympus) under 20x magnification. The images were processed using Mshot imaging software (MSX2).

### Neurite length analysis

The SH-SY5Y cells (1.5 × 10^5^ cells/mL) were cultured in T-25 flasks and treated accordingly. At the indicated days, the images of undifferentiated and differentiated cells with different serum concentrations were captured using CKX41 inverted microscope (Olympus) and the images were processed using Mshot imaging software (MSX2). The average neurite length of the undifferentiated and differentiated cells was analysed using NeuronJ plugin in ImageJ software and calculation were carried out using Excel sheet.

### Cholinergic markers gene expression study

The SH-SY5Y cells (1.5 × 10⁵ cells/mL) were cultured in T-75 flasks according to treatment conditions. At designated time points, undifferentiated and differentiated cells were harvested using a cell scrapper in 1X PBS (pH 7.4) and subjected to total RNA extraction (PrimeWay Total RNA Extraction Kit, KIT-9021). The RNA quantity and purity were assessed using a NanoPhotometer^®^ N60 (Implen, Germany), with all samples exhibiting acceptable absorbance ratios (A260/A280 = 1.80–2.00). RT-qPCR was performed using the qPCRBIO SyGreen 1-Step Go Detect Low-ROX kit (PCR Biosystems, CAT# PB25.11-01). Commercial primer sets (OligoMy, Malaysia) targeting choline acetyltransferase (*ChAT*), acetylcholinesterase (*AChE*), and glyceraldehyde 3-phosphate dehydrogenase (*GAPDH*) were used (Table S3). *GAPDH* served as the internal reference gene. The amplification protocol consisted of 40 cycles of denaturation at 95 °C for 5 s, followed by annealing at 60 °C (*AChE*) or 62 °C (*ChAT*) for 30 s. All reactions were performed in triplicate and melt curve analysis was conducted to confirm specificity using the QuantStudio™ 5 Real-Time PCR System. Relative gene expression was calculated using the 2^−ΔΔCt method (Rao et al., 2013).

### Acetylcholinesterase (AChE) activity

The AChE enzymatic activity was determined using a modified Ellman colorimetric method [15]. The SH-SY5Y cells were plated onto 96-well plate (1.5 × 10^5^ cells/mL) and treated accordingly. The spent media was removed, and the plates were washed with 1X PBS (pH 7.4) twice to remove any remaining media. Afterwards, ice-cold RIPA buffer (1X) (Abcam, USA) was added into each well and left to incubate on ice for 20–30 min. The cells were resuspended by pipetting. The reaction mixture containing Ellman regent (0.01 M) (ThermoFisher Scientific, USA), acetylcholine iodide (0.01 M) (ThermoFisher Scientific, USA), and 1X PBS (pH 7.4) (Sigma-Aldrich, USA) was added into each well. Afterwards, the absorbance readings were measured at 412 nm for 10 min at one-minute interval. The results were expressed in U/min/mL.

### Susceptibility of RA-differentiated SH-SY5Y to AD-related stressors in-vitro

The SH-SY5Y cells (1.5 × 10^5^ cells/mL) were seeded into 96-well plates and left to adhere for 24-hours. Followed by RA-induced differentiation in 1% HI-FBS for 7-days to produce cholinergic-like neurons. The cells were treated with two-fold dilutions of AD-related stressors namely, streptozotocin (STZ) (MedChemExpress, USA), lipopolysaccharide (LPS) (Santa Cruz Biotechnology, USA), aluminium chloride (AlCl_3_) (Bendosen, Malaysia), and hydrogen peroxide (H_2_O_2_) (Merck, Germany) prepared in low serum media (1% HI-FBS) and incubated for 24-, 48-, and 72-hour. Afterwards, CCK-8 (Dojindo, Japan) was added into each well and incubated for 3 h at 37 °C in a humidified atmosphere (5% CO_2_) incubator. The absorbance readings were measured at an excitation wavelength of 450 nm with a reference wavelength of 650 nm. Then cell viability was calculated based on the following formula.

$$\text{Cell Viability (\%)} = \frac{\mathrm{ABS(Treated)} - \mathrm{ABS(Blank)}}{\mathrm{ABS(Untreated)} - \mathrm{ABS(Blank)}} \times 100\%$$

### Statistical analysis

All experimentations were carried out in three independent experiments with three replicates. The results are presented as mean ± SEM using Microsoft Excel. The statistical significance was analysed using Statistical Package for the Social Sciences (SPSS, IBM) version 29 (ver. 29) using either One-Way Analysis of Variance (One-Way ANOVA) or Two-Way Analysis of Variance (Two-Way ANOVA) followed by Tukey’s Post-Hoc analysis to determine the significance difference between treatment groups. The *p*-*value < 0.05* is used as threshold of significance.

## Results

### Literature search on available differentiation protocols

**Fig. 2:**
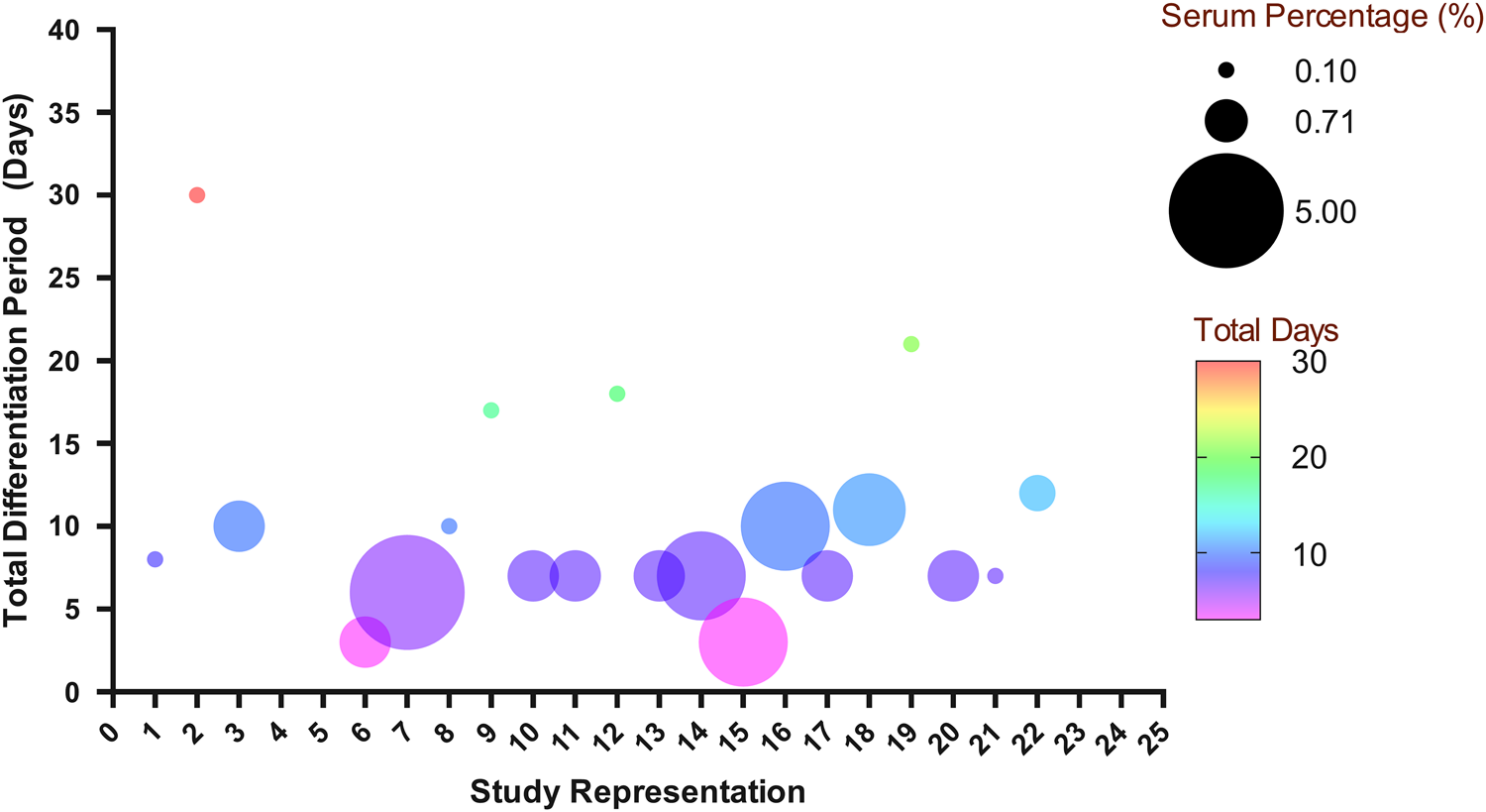
Summary bubble plot of compiled studies (2000–2024) available SH-SY5Y differentiation in literature. (A) The size of the bubble represents the concentration (%) of foetal bovine serum (FBS) used in each study while the gradient colour represents the total number of days used to differentiate the cells (B) On the X-axis shows the numerical representation of compiled studies. (C) On Y-axis represent the total number of days of differentiation protocol conducted based on the respective study

A total of 23 studies were included with differentiation protocols analysed based on serum concentration and maximum differentiation duration (Fig. 2 and Table S3). Considerable variability was observed across studies. Most studies employed retinoic acid (RA,10µM) combined with brain-derived neurotrophic factor (BDNF, 50ng/mL), except for six studies [19–24]. Serum concentrations ranged from 0% to 5% with the highest reported by Froster et al., [25], while several studies used serum-free conditions (0%) [9, 26–28]. However, some reports did not specify serum concentration [12, 23, 29–31]. Lastly, the differentiation periods varied from 3 days [19, 21] to 30 days [27].

Neuronal and cholinergic markers are summarised in Table S4. Nearly all studies (96%), except Arslan et al. (2020) assessed neuronal markers which confirms that SH-SY5Y cells differentiate into mature neuron-like cells irrespective of protocol variations. Frequently used markers included beta-tubulin III (βIII-tubulin) (52%), followed by microtubule-associated protein 2 (MAP2) (49%), synaptophysin 1 (SYP1) (26%), neuronal nuclei (NeuN) (13%), and synapsin 1 (Syn-1) (13%). In contrast, only 30% of studies evaluated cholinergic markers, primarily acetylcholinesterase (AChE) and choline acetyltransferase (ChAT) while choline transporter 1 (CHT-1) was less reported. All studies assessing cholinergic differentiation used RA+BDNF, except Ducray et al. (2020) [22], which used RA alone. Given that cholinergic dysfunction, characterised by reduced AChE and ChAT activity, is a hallmark of AD pathology, inclusion of cholinergic markers is particularly relevant. Notably, all seven studies reported significant upregulation of cholinergic markers following differentiation.

### Morphological Study

**Fig. 3:**
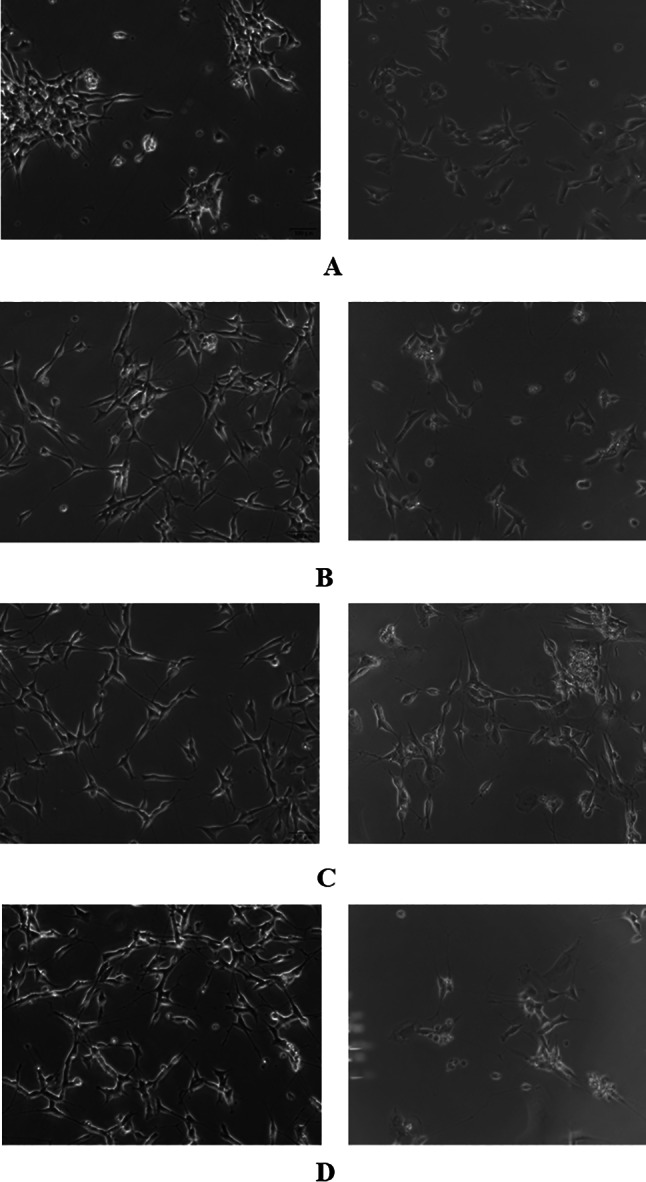
Morphological analysis of SH-SY5Y cells differentiation using 10 μm all-trans retinoic acid (RA) and different serum concentrations wherein left panel depicts 1% HI-FBS (DM1) and right panel depicts 3% HI-FBS (DM2). The time-points; (**A**) Day-0 (Undifferentiated); (**B**) Day-3; (**C**) Day-5 and; (**D**) Day-7 under 20x magnification. Day 0 represents the undifferentiated condition (without retinoic acid), whereas cells at Day 3, Day 5, and Day 7 were treated with 10µM all-trans retinoic acid

The undifferentiated SH-SY5Y cells appeared neuroblast-like characterized by large, flat, and non-polarized cell bodies with short, truncated neurite-like processes and larger soma. The cells grow in clusters and form clumps connected to adjacent cells with short neurites regardless of the serum concentrations at Day-0 (Fig. 3A). The SH-SY5Y were treated with two differentiation media containing either 1% or 3% HI-FBS in the presence of 10µM RA produced differentiated SH-SY5Y cells sharing similar characteristics to primary neurons. The differentiated cells have considerably longer and exquisite neurite process with extensive branching and becoming more polarized. The cells are also connected to adjacent neurons extensively with increasing differentiation periods; 3-day, 5-day, and 7-day (Fig. 3B-D). Decreasing the serum concentrations with longer differentiation period shows a decrease in cell density. However, differentiation media containing 1% HI-FBS produced larger population of mature neuron-like cells observed by prominent, longer, and defined neurite projections. These cells also possess extensive neurite branching and connected to far more adjacent neurons across all time-points compared to undifferentiated SH-SY5Y (Day-0).

### Neurite length analysis

**Fig. 4:**
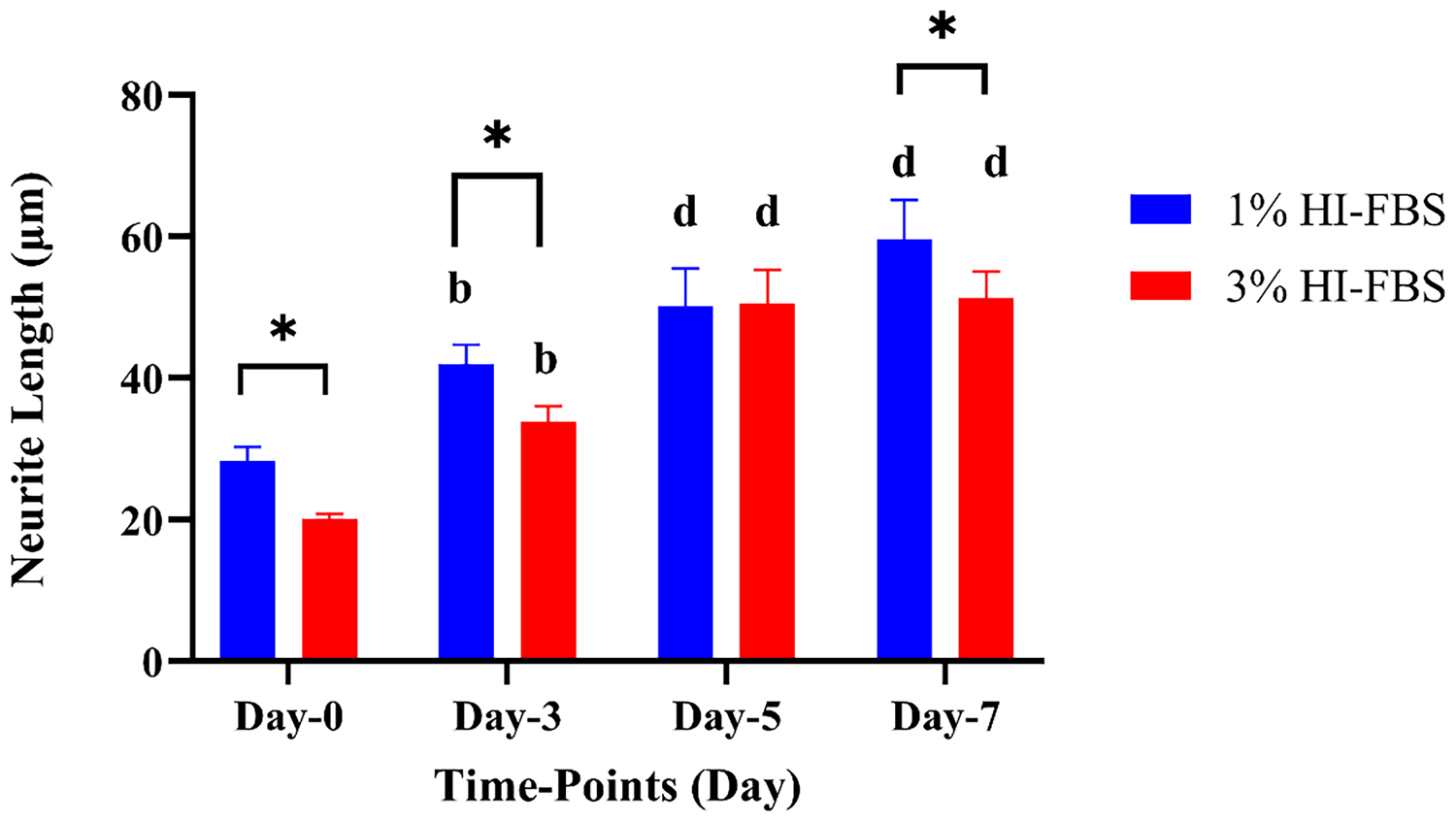
Neurite length analysis of differentiated SH-SY5Y in different serum concentrations of 1% HI-FBS (DM1) or 3% HI-FBS (DM2) and differentiation periods (Day-0, Day-3, Day-5, Day-7). Day 0 represents the undifferentiated condition (without retinoic acid), whereas cells at Day 3, Day 5, and Day 7 were treated with 10µM all-trans retinoic acid. The results are represented by mean ± SEM of three independent experiments (*n* = 3) in triplicates. The statistical analysis was carried out using Two-Way ANOVA followed by Tukey’s post-hoc analysis (a*p < 0.05, b**p < 0.01, c***p < 0.001, d****p < 0.0001). The letters denote comparison between treated groups with undifferentiated condition (without retinoic acid), while the asterisks denote comparison between serum concentration (%)

The average neurite length of undifferentiated SH-SY5Y (Day-0) in 1% and 3% HI-FBS were 28.28 ± 1.18 μm and 20.17 ± 0.40 μm, respectively. The differentiated cells in both differentiation media exhibited markedly longer neurites compared to undifferentiated SH-SY5Y with increasing time-points (Fig. 4). Like the morphological study, it showed that SH-SY5Y differentiated in 1% HI-FBS condition expressed longer neurite length with average length of 41.97 ± 1.59 μm (*p* < 0.01), 50.18 ± 3.07 μm (*p* < 0.0001), and 59.60 ± 3.23 μm (*p* < 0.0001) at Day-3, Day-5, and Day-7, respectively. While in 3% HI-FBS showed relatively shorter average neurite length with 33.82 ± 1.29 μm (*p* < 0.0001), 50.41 ± 2.81 μm (*p* < 0.0001), and 51.30 ± 2.17 μm (*p* < 0.0001) across the time-points. Interestingly, at two serum concentrations (1% HI vs. 3% HI), the average neurite length was considered insignificant at Day-5. While at Day-3 and Day-7 were considered statistically significant (*p* < 0.05).

### RT-qPCR analysis of cholinergic markers

**Fig. 5:**
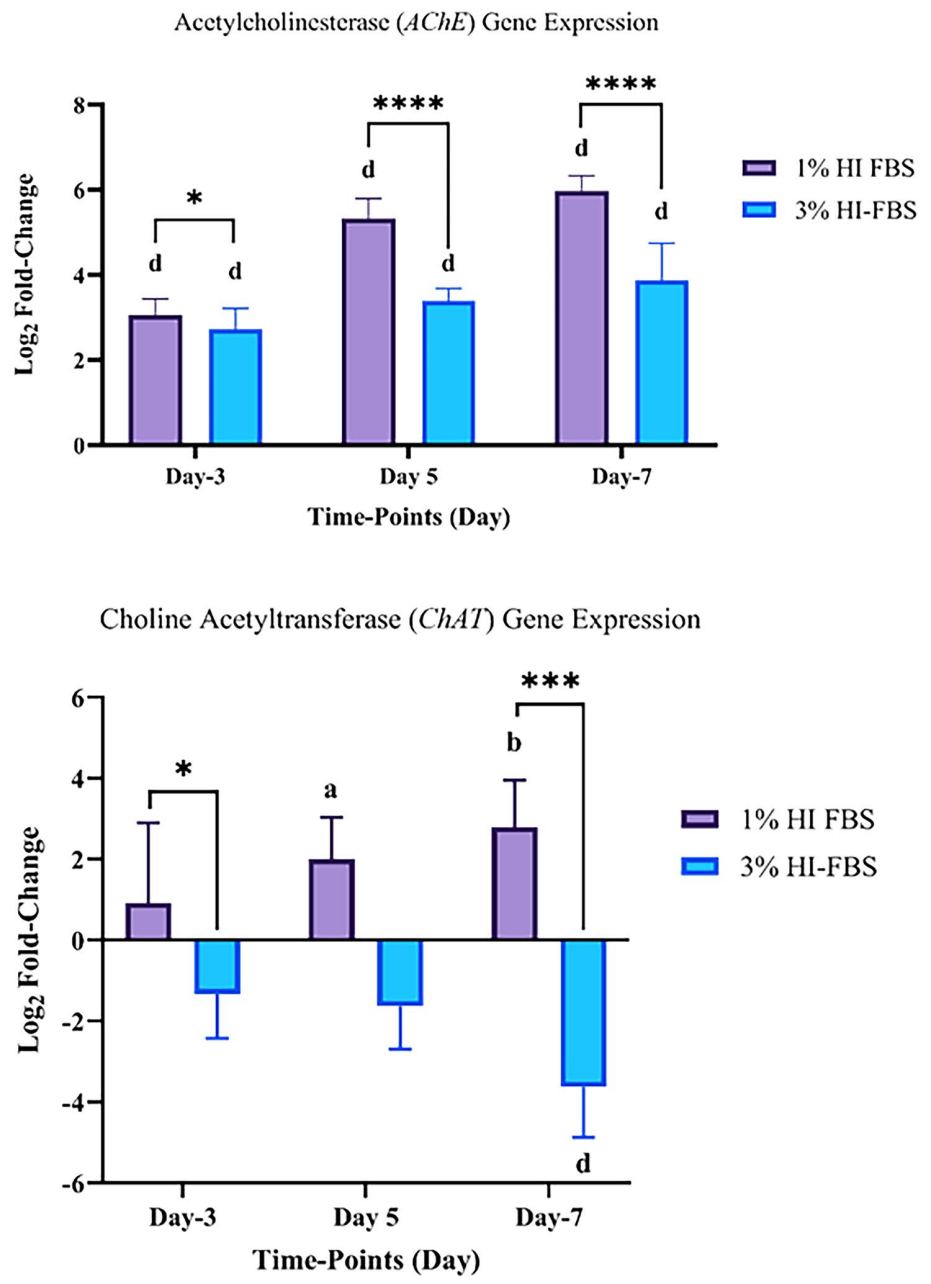
Reverse transcription quantitative PCR (RT-qPCR) analysis of cholinergic markers in differentiated SH-SY5Y in different serum concentrations of 1% HI-FBS (DM1) or 3% HI-FBS (DM2) and differentiation periods (Day-0, Day-3, Day-5, Day-7). Day 0 represents the undifferentiated condition (without retinoic acid), whereas cells at Day 3, Day 5, and Day 7 were treated with 10µM all-trans retinoic acid. Histogram showing the base 2 logarithm fold-change (Log2 FC) of qPCR analysis of two cholinergic markers; (A) Acetylcholinesterase (*AChE*) and (B) Choline Acetyltransferase (*ChAT*) after normalizing using GAPDH. Results are expressed log_2_ fold-change compared to undifferentiated cells (Day-0) which correspond to log_2_ fold-change of 0 (baseline; not shown). The results are represented by mean ± SEM of three independent experiments (*n* = 3) in triplicates. The statistical analysis was carried out using Two-Way ANOVA followed by Tukey’s post-hoc analysis (a*p < 0.05, b**p < 0.01, c***p < 0.001, d****p < 0.0001). The letters denote comparison between treated groups with undifferentiated condition (without retinoic acid), while the asterisks denote comparison between serum concentration (%)

The relative log_2_ fold-change (Log_2_ FC) expression of cholinergic markers; acetylcholinesterase (*AChE*) and choline acetyltransferase (*ChAT*) in differentiated SH-SY5Y were expressed in relative to expression observed in undifferentiated SH-SY5Y cells. In both 1% and 3% HI-FBS differentiation conditions, it was significantly upregulated *AChE* expression across all time-points (Fig. 5). It shows 1% HI-FBS it produced more prominent and significant upregulation of *AChE* as its expression increased 3.05-fold (*p* < 0.0001), 5.31-fold (*p* < 0.0001), and 5.69-fold (*p* < 0.0001) at Day-3, Day-5, and Day-7 respectively compared to undifferentiated SH-SY5Y (Log_2_ FC = 0, baseline). Similarly, in 3% HI-FBS although it significantly increased *AChE* expression by 2.7-fold (*p* < 0.0001), 3.39-fold (*p* < 0.0001), and 3.87-fold (*p* < 0.0001) across the time-points than undifferentiated cells (Log_2_ FC = 0, baseline). However, 1% HI-FBS differentiation media produced far more prominent upregulation of *AChE* expression compared to 3% HI-FBS at Day-5 (*p* < 0.0001) and Day-7 (*p* < 0.0001) while at Day-3 showed minimal difference (*p* < 0.05). In 1% HI-FBS produced significant upregulation of *ChAT* expression at Day-5 and Day-7 with 2.0-fold (*p* < 0.05) and 2.8-fold (*p* < 0.01), respectively. However, in 3% HI-FBS showed significant downregulation of *ChAT* expression of -3.6-fold change (*p* < 0.0001) at Day-7, but there were insignificant reductions in *ChAT* expression of -1.3-fold and − 1.6-fold change at Day-3 and Day-5. At Day-7, it caused significant downregulation of *ChAT* expression.

### Acetylcholinesterase (AChE) activity

**Fig. 6:**
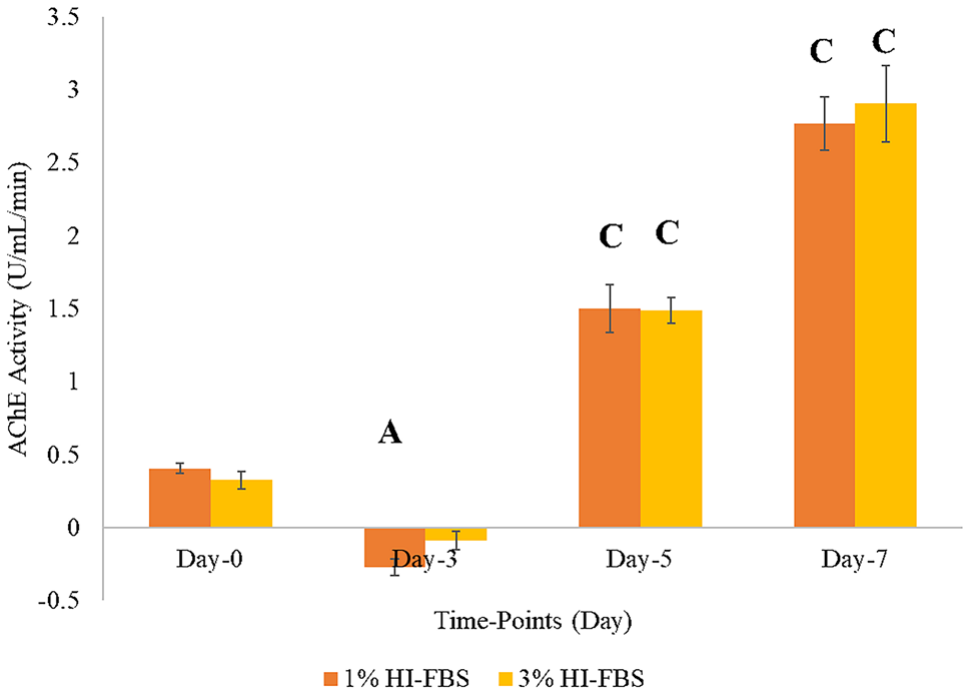
Acetylcholinesterase (AChE) activity of RA-differentiated SH-SY5Y in different serum concentrations of 1% HI-FBS (DM1) or 3% HI-FBS (DM2) at four time-points; Day-0, Day-3, Day-5, Day-7. Day 0 represents the undifferentiated condition (without retinoic acid), whereas cells at Day 3, Day 5, and Day 7 were treated with 10µM all-trans retinoic acid. The acetylcholinesterase activity was performed using Ellman reagent and measured at 412 nm for 10 min at 1-minute intervals. The results are represented by mean ± SEM of three independent experiments (*n* = 3) in triplicates. The statistical analysis was carried out using Two-Way ANOVA followed by Tukey’s post-hoc analysis (A*p < 0.05, B**p < 0.01, C***p < 0.001). The letters denote comparison between treated groups with undifferentiated condition (without retinoic acid), while the asterisks denote comparison between serum concentration (%)

The acetylcholinesterase (AChE) activity was measured using the Ellman method on RA-differentiated SH-SY5Y using different serum concentrations (%) and time-points (Fig. 6). In both differentiation media, it shows that AChE activity had a time-dependent response. It showed a marked decrease in AChE activity of -2.68 ± 0.099 U/mL/min (*p* < 0.05) when differentiated for 3-days in 1% HI-FBS while in 3% HI-FBS was an insignificant drop of activity of -0.87 ± 0.111 U/mL/min. Notably, extended differentiation period to 5 and 7 days resulted in a significant increase in AChE activity. At Day-5, there was an increased in AChE activity of 1.50 ± 0.611 U/mL/min (*p* < 0.001) and 1.49 ± 0.157 U/mL/min (*p* < 0.001) for 1% and 3% HI-FBS, respectively. Across all time-points, Day-7 had the highest observed AChE activity with 2.77 ± 0.316 U/mL/min (*p* < 0.001) and 2.90 ± 0.454 U/min/mL (*p* < 0.001) when differentiated with 1% and 3% HI-FBS, respectively. However, there was insignificant difference between the serum concentration used to AChE activity.

### Susceptibility of cholinergic-like neurons to AD-related stressors in-vitro

**Fig. 7:**
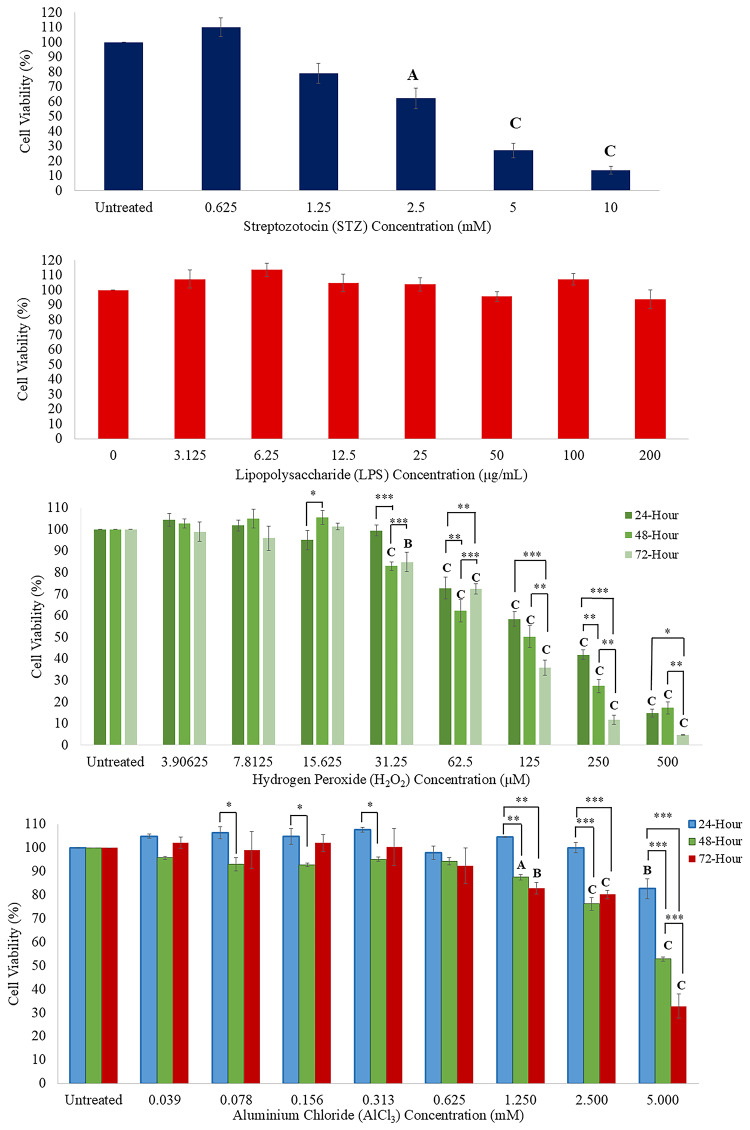
In-vitro cytotoxicity study using CCK-8 cell viability assay for susceptibility of cholinergic-like neurons produced from 7-day differentiation of SH-SY5Y in 1% HI-FBS with 10µM RA to AD-related stressors. The cholinergic-like neurons were treated with four AD-related stressors. (**A**) streptozotocin (STZ), (**B**) lipopolysaccharide (LPS), (**C**) hydrogen peroxide (H_2_O_2_), and (D) aluminium chloride (AlCl_3_) at three different time-points (24-, 48-, 72-hour), whereas STZ and LPS treatments were limited 24-hour exposure due to stability limitations and the lack of appropriate surface markers, respectively, for extended assessment. The results are represented by mean ± SEM of three independent experiments (*n* = 3) in triplicates. The statistical analysis was carried out using either One-Way ANOVA or Two-Way ANOVA followed by Tukey’s post-hoc analysis (A*p < 0.05, B**p < 0.01, C***p < 0.001, D****p < 0.0001). The letters denote comparison between treated groups with untreated control while the asterisks denote comparison between time-points

This study also investigated the susceptibility of these cholinergic-like neurons to four AD-related stressors such as streptozotocin (STZ), lipopolysaccharide (LPS), aluminium chloride (AlCl_3_), and hydrogen peroxide (H_2_O_2_) for in-vitro models using CCK-8 cell viability assay (Fig. 7.). All stressors, except LPS, exhibited a dose-dependent response, whereas only AlCl₃ and H₂O₂ demonstrated a time-dependent effect. For STZ (Fig. 7A) showed significant reduction in cell viability at concentration of 2.5mM – 10mM with an IC_50_ (mM) of 4.27 ± 0.210. Interestingly, no significant reduction in cell viability was observed in LPS-treated group despite increasing the concentration to 200 µg/mL indicating that these cholinergic neurons are non-susceptible to LPS-elicit cytotoxicity as the cell viability maintained at 90% across the concentration range (Fig. 7B). For H_2_O_2_ exhibited both dose- and time-dependent responses (Fig. 7.C). At 24 h, significant cytotoxicity was observed at 62.5µM, while at 48 and 72 h, reduced cell viability was evident at 31.25µM. The IC_50_ for H_2_O_2_ were determined to be 134.44 ± 3.48µM, 117.00 ± 3.19µM, and 74.26 ± 0.75µM for 24-hour, 48-hour, and 72-hour. Similarly, AlCl_3_ showed dose- and time-dependent responses (Fig. 7D). In the 24-hour time-point, it shows that AlCl_3_ did not elicit cytotoxicity to the cholinergic-like neurons all concentrations, however, the cell viability dropped to 82.5% when treated with 5mM of AlCl_3_. While at 48- and 72-hour, it showed a significant reduction in cell viability to 87.5% and 82.2% at concentration of 1.25mM, respectively. The IC_50_ of AlCl_3_ was determined to be 7.49 ± 1.42mM and 3.84 ± 0.30mM for 48-hour and 72-hour time-point only.

## Discussion

It highlighted that RA alone or in combination BDNF consistently induces structural maturation with differentiated SH-SY5Y exhibit extensive neurite branching, elongation, and neuron-like morphology [19–30, 32–37]. Noteworthily, serum deprivation (0–1%) is frequently employed during RA differentiation [8–9, 15, 26–19, 27, 33–28, 34, 36–22, 24]. This is due to presence of N-type and S-type population [38, 39]. Wherein higher serum concentrations (10% RA/TPA) promote S-type proliferation and reduce neuronal-specific population [17]. In contrast, reduced serum enhances N-type differentiation and promote S-type apoptosis, yielding a more homogenous neuronal population [15, 17]. In the present study, 10µM RA in 1% or 3% HI-FBS induced consistent neuron-like morphological changes across time-points (Figs. 3 and 4) characterised by extensive neurite projections, in agreement with previous reports [27, 28, 31, 36]. Although mature neuronal markers were not assessed, Nasir et al., (2024) [40] that RA-differentiation of SH-SY5Y with 1% or 3% FBS with 10µM RA for 7 days significantly increased βIII-tubulin intensity and positively correlating with dendritic length. supporting neuronal maturation. Collectively, these findings suggest that 1% HI-FBS with 10µM is sufficient in generating a relatively mature neuronal-like population suitable for high-throughput in-vitro neurodegenerative studies.

Differentiation of SH-SY5Y cells lacks consistent subtype characterisation, as their phenotype depends on differentiation conditions which may develop into cholinergic, dopaminergic, adrenergic, or glutamatergic subtypes [11]. Only 30% of studies assess markers such as ChAT and AChE [8, 15, 22, 25–33]. Most employ 10µM RA with 50ng/mL BDNF under varying serum conditions, except Ducary et al., 2020 [22], who used RA alone. RA regulates 28% of neuronal differentiation-related genes, promoting neurite outgrowth, synaptic activity, and growth arrest [12, 13]. As TrkB mediates BDNF signalling, RA–BDNF synergy can enhance cholinergic differentiation [15]. Indeed, RA+BDNF increased ChAT and AChE activities compared with RA alone [15], whereas Ducary et al. (2020) [22] reported RA alone induced the highest CHT1 expression. Given the vulnerability of cholinergic neurons in AD and their role in cognition [15], inclusion of cholinergic markers is essential for in vitro AD models.

In this study, serum concentration and differentiation duration in presence of retinoic acid alone significantly influenced cholinergic gene expression. Both 1% and 3% HI-FBS upregulated *AChE* mRNA, with stronger effect at 1% (Fig. 5) which is consistent with previous reports [15, 25, 31, 33]. Conversely, 3% HI-FBS downregulated *ChAT* expression at all time-points, whereas 1% HI-FBS increased its expression similarly reported in literature [8]. RA in serum-free conditions have been shown to elevate *ChAT*,* AChE*, and *VAChT* via protein kinase C (PKC)-mediated mechanisms in differentiated NG108-15 [41, 42]. Hence, serum deprivation to 1% is required to upregulate cholinergic marker gene expressions in producing cholinergic phenotype for in-vitro AD model. Although *AChE* m RNA was significantly increased at Day-3, enzymatic activity was reduced under 1% HI-FBS and slightly decreased under 3% HI-FBS (Fig. 6) which suggest transcriptional activation precedes functional AChE expression. A similar observation was reported in P19 cells differentiation whereby *AChE* mRNA was detected without measurable AChE activity during early differentiation [43]. These findings indicate that serum deprivation to 1% and longer differentiation period enhances cholinergic gene expression and supports induction of a cholinergic phenotype in RA-differentiated SH-SY5Y. Additionally, protein-level validation such as immunocytochemistry and Western blot is warranted in corroborating these observations and cholinergic population homogeneity. Nonetheless, RA-only differentiation with 1% HI-FBS is sufficient to induce cholinergic-like features in SH-SY5Y cells, as evidenced by the upregulation of cholinergic-associated markers, without the need for additional factors such as BDNF, thereby offering a simpler and more cost-effective approach.

The cholinergic-like neurons exhibited a dose-dependent response whereas LPS produced no observable toxicity (Fig. 7B). RA-differentiated SH-SY5Y cells express low levels of TLR3/4, hence limiting responsiveness to LPS and downstream pro-inflammatory signalling [44] Consistently, LPS cytotoxicity is typically observed in microglial co-culture or primed models [5], indicating limited suitability in neuronal monoculture systems. STZ induced dose-dependent cytotoxicity (Fig. 7A) which consistent with reports in multiple neuronal cell lines [45]. Its uptake via GLUT2 and intracellular conversion to diazomethane (DAM) promotes DNA damage and oxidative stress [45]. Although time-dependent effects have been reported [46], exposure was limited to 24 h in this study due to STZ instability [45]. AlCl_3_ did not induced significant cytotoxicity at 24-hour (Fig. 7D), likely due to its lipophobicity and limited intracellular accumulation. More lipophilic aluminium complexes exhibit greater neurotoxicity [47, 48], while RA-differentiated SH-SY5Y cells demonstrate relative resistance to aluminium insults, hence require longer incubation period and higher concentration to elicit cytotoxicity [27]. Similarly, H_2_O_2_ elicited dose- and time-dependent cytoxicity (Fig. 7C) corroborating its role in inducing oxidative stress via mitochondrial dysfunction and ROS generation [49]. Although RA differentiation enhances antioxidant defences (e.g. SOD, CAT), however at higher concentration or prolonged exposure to H_2_O_2_, it may overwhelm these systems and promote mitochondrial-mediated apoptosis [50].

The RA-differentiated SH-SY5Y model presents several strengths, including its methodological simplicity, cost-effectiveness, and reproducibility, particularly, through the use of a single inducers (RA) under low-serum (1%) condition in generating neuronal-like cells with cholinergic-like phenotype. The model demonstrates functional relevance by exhibiting upregulation of key cholinergic markers (*ChAT* and *AChE*) and responsiveness to multiple AD-associated stressors, supporting its suitability for mechanistic and pharmacological studies. However. limitations include the incomplete recapitulation of primary basal forebrain cholinergic neuron complexity, relatively low representation of fully mature cholinergic features compared to in-vivo systems, and lack of inflammatory responsiveness, which may restrict its utility in modelling neuroinflammation aspects in AD. Nonetheless, the future applications of this model include its use a high-throughput screening platform for cholinergic-targeting therapeutics, investigation of oxidative stress-mediated neurodegenerations, and preliminary validation of neuroprotective compounds prior to complex in-vitro and in-vivo models.

## Conclusion

The inconsistency among available differentiation protocols, including variations in serum concentrations, differentiation inducers, and differentiation durations, has complicated the generation of neuronal-like cells with cholinergic-like characteristics from RA-differentiated SH-SY5Y cells. In this study, we provide further evidence that differentiation using 1% HI-FBS for 7 days in the presence of 10µM RA can reliably generate mature neuronal-like cells exhibiting a cholinergic-like phenotype, as demonstrated by significant upregulation of *AChE* and *ChAT* gene expressions, accompanied by increased in AChE activity compared with undifferentiated cells. These cholinergic-like neurons were susceptible to STZ-, H_2_O_2_-, and AlCl_3_-induced cytotoxicity, whereas LPS was found to be non-cytotoxic. Overall, these findings highlight the potential applicability of this cell model in AD research. Furthermore, the proposed model may serve as a convenient platform for high-throughput screening of neuroprotective against neurotoxin-induced AD-like pathologies in-vitro.

## Supplementary Information

Below is the link to the electronic supplementary material.

Supplementary Material 1

## Data Availability

The datasets generated and/or analysed during the current study are available from the corresponding author upon reasonable request.

## References

[1] Buccellato FR, D’Anca M, Tartaglia GM, Del Fabbro M, Scarpini E, Galimberti D (2023) Treatment of Alzheimer’s Disease: Beyond Symptomatic Therapies. Int J Mol Sci 24(18):13900. 10.3390/ijms241813900 37762203 10.3390/ijms241813900 PMC10531090

[2] World Health Organization (WHO), Ageing and health, World Health Organization (WHO) (2024) https://www.who.int/news-room/fact-sheets/detail/ageing-and-health (Accessed 2024-10-21)

[3] Parums DV (2024) A Review of the Current Status of Disease-Modifying Therapies and Prevention of Alzheimer’s Disease. Med Sci Monit 30:e945091. 10.12659/MSM.945091 38736218 10.12659/MSM.945091 PMC11097689

[4] Cummings J, Zhou Y, Lee G, Zhong K, Fonseca J, Cheng D (2024) Alzheimer’s disease drug development pipeline: 2024. Alzheimers Dement (NY) 10(2):e12465. 10.1002/trc2.12465 10.1002/trc2.12465 PMC11040692 38659717

[5] Liu PP, Xie Y, Meng XY, Kang JS (2019) History and progress of hypotheses and clinical trials for Alzheimer’s disease. Sig Transduct Target Ther 4:29. 10.1038/s41392-019-0063-8 10.1038/s41392-019-0063-8 PMC6799833 31637009

[6] Zhang J, Zhang Y, Wang J et al (2024) Recent advances in Alzheimer’s disease: mechanisms, clinical trials, and new drug development strategies. Sig Transduct Target Ther 9:211. 10.1038/s41392-024-01911-3 10.1038/s41392-024-01911-3 PMC11344989 39174535

[7] Stanciu GD, Luca A, Rusu RN, Bild V, Beschea Chiriac SI, Solcan C, Bild W, Ababei DC (2019) Alzheimer’s Disease Pharmacotherapy in Relation to Cholinergic System Involvement. Biomolecules 10(1):40. 10.3390/biom10010040 31888102 10.3390/biom10010040 PMC7022522

[8] Targett IL, Crompton LA, Conway ME, Craig TJ (2024) Differentiation of SH-SY5Y neuroblastoma cells using retinoic acid and BDNF: a model for neuronal and synaptic differentiation in neurodegeneration. Vitro Cell Dev Biol Anim 60(9):1058–1067. 10.1007/s11626-024-00948-6 10.1007/s11626-024-00948-6 PMC11534981 39017752

[9] Shipley MM, Mangold CA, Szpara ML (2016) Differentiation of the SH-SY5Y Human Neuroblastoma Cell Line. J Vis Exp 17(108):53193. 10.3791/53193 10.3791/53193 PMC4828168 26967710

[10] Xicoy H, Wieringa B, Martens GJ (2017) The SH-SY5Y cell line in Parkinson’s disease research: a systematic review. Mol Neurodegeneration 12:10. 10.1186/s13024-017-0149-0 10.1186/s13024-017-0149-0 PMC5259880 28118852

[11] Bell M, Zempel Hans (2022) SH-SY5Y-derived neurons: a human neuronal model system for investigating TAU sorting and neuronal subtype-specific TAU vulnerability. Rev Neurosci 33(1):1–15. 10.1515/revneuro-2020-0152 33866701 10.1515/revneuro-2020-0152

[12] Teppola H, Sarkanen JR, Jalonen TO, Linne ML (2016) Morphological Differentiation Towards Neuronal Phenotype of SH-SY5Y Neuroblastoma Cells by Estradiol, Retinoic Acid and Cholesterol. Neurochem Res 41(4):731–747. 10.1007/s11064-015-1743-6 26518675 10.1007/s11064-015-1743-6 PMC4824837

[13] Kalinovskii AP, Osmakov DI, Koshelev SG, Lubova KI, Korolkova YV, Kozlov SA, Andreev YA (2022) Retinoic Acid-Differentiated Neuroblastoma SH-SY5Y Is an Accessible In Vitro Model to Study Native Human Acid-Sensing Ion Channels 1a (ASIC1a). Biology 11(2):167. 10.3390/biology11020167 35205034 10.3390/biology11020167 PMC8868828

[14] Feles S, Overath C, Reichardt S, Diegeler S, Schmitz C, Kronenberg J, Baumstark-Khan C, Hemmersbach R, Hellweg CE, Liemersdorf C (2022) Streamlining Culture Conditions for the Neuroblastoma Cell Line SH-SY5Y: A Prerequisite for Functional Studies. Methods Protoc 5(4):58. 10.3390/mps5040058 35893584 10.3390/mps5040058 PMC9326679

[15] de Medeiros LM, De Bastiani MA, Rico EP, Schonhofen P, Pfaffenseller B, Wollenhaupt-Aguiar B, Grun L, Barbé-Tuana F, Zimmer ER, Castro MAA, Parsons RB, Klamt F (2019) Cholinergic Differentiation of Human Neuroblastoma SH-SY5Y Cell Line and Its Potential Use as an In vitro Model for Alzheimer’s Disease Studies. Mol Neurobiol 56(11):7355–7367. 10.1007/s12035-019-1605-3 31037648 10.1007/s12035-019-1605-3

[16] Martin ER, Gandawijaya J, Oguro-Ando A (2022) A novel method for generating glutamatergic SH-SY5Y neuron-like cells utilizing B-27 supplement. Front Pharmacol 20(13):943627. 10.3389/fphar.2022.943627 10.3389/fphar.2022.943627 PMC9630362 36339621

[17] Magalingam KB, Radhakrishnan AK, Somanath SD et al (2020) Influence of serum concentration in retinoic acid and phorbol ester induced differentiation of SH-SY5Y human neuroblastoma cell line. Mol Biol Rep 47:8775–8788. 10.1007/s11033-020-05925-2 33098048 10.1007/s11033-020-05925-2

[18] Khazeem MM, Casement JW, Schlossmacher G, Kenneth NS, Sumbung NK, Chan JYT, McGow JF, Cowell IG, Austin CA (2022) TOP2B Is Required to Maintain the Adrenergic Neural Phenotype and for ATRA-Induced Differentiation of SH-SY5Y Neuroblastoma Cells. Mol Neurobiol 59(10):5987–6008. 10.1007/s12035-022-02949-6 35831557 10.1007/s12035-022-02949-6 PMC9463316

[19] Simões RF, Ferrão R, Silva MR, Pinho SLC, Ferreira L, Oliveira PJ, Cunha-Oliveira T (2021) Refinement of a differentiation protocol using neuroblastoma SH-SY5Y cells for use in neurotoxicology research. Food Chem Toxicol 149:111967. 10.1016/j.fct.2021.111967 33417974 10.1016/j.fct.2021.111967

[20] Cheung YT, Lau WK, Yu MS, Lai CS, Yeung SC, So KF, Chang RC (2009) Effects of all-trans-retinoic acid on human SH-SY5Y neuroblastoma as in vitro model in neurotoxicity research. Neurotoxicology 30(1):127–135. 10.1016/j.neuro.2008.11.001 19056420 10.1016/j.neuro.2008.11.001

[21] Dwane S, Durack E, Kiely PA (2013) Optimising parameters for the differentiation of SH-SY5Y cells to study cell adhesion and cell migration. BMC Res Notes 11:6:366. 10.1186/1756-0500-6-366 10.1186/1756-0500-6-366 PMC3847106 24025096

[22] Ducray AD, Wiedmer L, Herren F, Widmer HR, Mevissen M (2020) Quantitative Characterization of Phenotypical Markers After Differentiation of SH-SY5Y Cells. CNS Neurol Disord Drug Targets 19(8):618–629. 10.2174/1871527319666200708132716 32640966 10.2174/1871527319666200708132716

[23] Filograna R, Civiero L, Ferrari V, Codolo G, Greggio E, Bubacco L, Beltramini M, Bisaglia M (2015) Analysis of the Catecholaminergic Phenotype in Human SH-SY5Y and BE(2)-M17 Neuroblastoma Cell Lines upon Differentiation. PLoS ONE 10(8):e0136769. 10.1371/journal.pone.0136769 26317353 10.1371/journal.pone.0136769 PMC4552590

[24] Hromadkova L, Bezdekova D, Pala J, Schedin-Weiss S, Tjernberg LO, Hoschl C, Ovsepian SV (2020) Brain-derived neurotrophic factor (BDNF) promotes molecular polarization and differentiation of immature neuroblastoma cells into definitive neurons. Biochim Biophys Acta Mol Cell Res 1867(9):118737. 10.1016/j.bbamcr.2020.118737 32389647 10.1016/j.bbamcr.2020.118737

[25] Forster JI, Köglsberger S, Trefois C, Boyd O, Baumuratov AS, Buck L, Balling R, Antony PM (2016) Characterization of Differentiated SH-SY5Y as Neuronal Screening Model Reveals Increased Oxidative Vulnerability. J Biomol Screen 21(5):496–509. 10.1177/1087057115625190 26738520 10.1177/1087057115625190 PMC4904349

[26] Jämsä A, Hasslund K, Cowburn RF, Bäckström A, Vasänge M (2004) The retinoic acid and brain-derived neurotrophic factor differentiated SH-SY5Y cell line as a model for Alzheimer’s disease-like tau phosphorylation. Biochem Biophys Res Commun 319(3):993–1000. 10.1016/j.bbrc.2004.05.075 15184080 10.1016/j.bbrc.2004.05.075

[27] Encinas M, Iglesias M, Liu Y, Wang H, Muhaisen A, Ceña V, Gallego C, Comella JX (2000) Sequential treatment of SH-SY5Y cells with retinoic acid and brain-derived neurotrophic factor gives rise to fully differentiated, neurotrophic factor-dependent, human neuron-like cells. J Neurochem 75(3):991–1003. 10.1046/j.1471-4159.2000.0750991.x 10936180 10.1046/j.1471-4159.2000.0750991.x

[28] Voogd EJHF, Doorn N, Levers MR, Hofmeijer J, Frega M (2024) Degree of differentiation impacts neurobiological signature and resistance to hypoxia of SH-SY5Y cells. J Neural Eng 20(6). 10.1088/1741-2552/ad17f3 10.1088/1741-2552/ad17f3 38128130

[29] Jahn K, Wieltsch C, Blumer N, Mehlich M, Pathak H, Khan AQ, Hildebrandt H, Frieling H (2017) A cell culture model for investigation of synapse influenceability: epigenetics, expression and function of gene targets important for synapse formation and preservation in SH-SY5Y neuroblastoma cells differentiated by retinoic acid. J Neural Transm (Vienna) 124(11):1341–1367. 10.1007/s00702-017-1769-9 28887651 10.1007/s00702-017-1769-9

[30] D’Aloia A, Pastori V, Blasa S, Campioni G, Peri F, Sacco E, Ceriani M, Lecchi M, Costa B (2024) A new advanced cellular model of functional cholinergic-like neurons developed by reprogramming the human SH-SY5Y neuroblastoma cell line. Cell Death Discov 10(1):24. 10.1038/s41420-023-01790-7 38216593 10.1038/s41420-023-01790-7 PMC10786877

[31] Pulkrabkova L, Muckova L, Hrabinova M, Sorf A, Kobrlova T, Jost P, Bezdekova D, Korabecny J, Jun D, Soukup O (2023) Differentiated SH-SY5Y neuroblastoma cells as a model for evaluation of nerve agent-associated neurotoxicity. Arch Toxicol 97(8):2209–2217. 10.1007/s00204-023-03525-0 37221426 10.1007/s00204-023-03525-0

[32] Sert SB, Tuğba E, Ergür, Uğur B, Pınar A (2020) Koçtürk semra comparison of medium supplements in terms of the effects on the differentiation of sh-sy5y human neuroblastoma cell line. Neurol Sci Neurophysiol 37(2):82–88. 10.4103/NSN.NSN_15_20

[33] Pereira ME, Lima LS, Souza JV, de Souza da Costa N, da Silva JF, Guiloski IC, Irioda AC, Oliveira CS (2024) Evaluation of the Neuroprotective Effect of Organic Selenium Compounds: An in Vitro Model of Alzheimer’s Disease. Biol Trace Elem Res 202(7):2954–2965. 10.1007/s12011-023-03893-9 37803188 10.1007/s12011-023-03893-9

[34] Moreira NCDS, Tamarozzi ER, Lima JEBF, Piassi LO, Carvalho I, Passos GA, Sakamoto-Hojo ET (2022) Novel Dual AChE and ROCK2 Inhibitor Induces Neurogenesis via PTEN/AKT Pathway in Alzheimer’s Disease Model. Int J Mol Sci 23(23):14788. 10.3390/ijms232314788 36499116 10.3390/ijms232314788 PMC9737254

[35] Dravid A, Raos B, Svirskis D, O’Carroll SJ (2021) Optimised techniques for high-throughput screening of differentiated SH-SY5Y cells and application for neurite outgrowth assays. Sci Rep 11(1):23935. 10.1038/s41598-021-03442-1 34907283 10.1038/s41598-021-03442-1 PMC8671469

[36] Alaylıoğlu M, Keskin E, Yediel BŞ, Dursun E, Ak DG (2024) A Novel and Robust Protocol for Differentiation of SH-SY5Y Neuroblastoma Cells into Neuron-Like Cells. Noro Psikiyatr Ars 61(3):208–212. 10.29399/npa.28510 39258131 10.29399/npa.28510 PMC11382563

[37] Arslan ME, Türkez H, Mardinoğlu A (2020) In vitro neuroprotective effects of farnesene sesquiterpene on alzheimer’s disease model of differentiated neuroblastoma cell line. Int J Neurosci 131(8):745–754. 10.1080/00207454.2020.1754211 32308094 10.1080/00207454.2020.1754211

[38] Bell N, Hann V, Redfern CP, Cheek TR (2013) Store-operated Ca (2+) entry in proliferating and retinoic acid-differentiated N- and S-type neuroblastoma cells. Biochim Biophys Acta 1833(3):643–651. 10.1016/j.bbamcr.2012.11.025 23220046 10.1016/j.bbamcr.2012.11.025 PMC3776921

[39] Corey JM, Gertz CC, Sutton TJ, Chen Q, Mycek KB, Wang BS, Martin AA, Johnson SL, Feldman EL (2010) Patterning N-type and S-type neuroblastoma cells with Pluronic F108 and ECM proteins. J Biomed Mater Res A 93(2):673–686. 10.1002/jbm.a.32485 19609877 10.1002/jbm.a.32485 PMC2845720

[40] Mohamad Nasir NF, Mohd Hazli MSH, Shamsuddin S et al (2024) Alteration of mature neuronal marker of β-III tubulin expression in differentiated SH-SY5Y cells by refinement of foetal bovine serum concentration. Beni-Suef Univ J Basic Appl Sci 13(84). 10.1186/s43088-024-00547-0

[41] Castell X, Diebler MF, Tomasi M, Bigari C, De Gois S, Berrard S, Mallet J, Israël M, Dolezal V (2002) More than one way to toy with ChAT and VAChT. J Physiol Paris 96(1–2):61–72. 10.1016/s0928-4257(01)00081-x 11755784 10.1016/s0928-4257(01)00081-x

[42] Leli U, Shea TB, Cataldo A, Hauser G, Grynspan F, Beermann ML, Liepkalns VA, Nixon RA, Parker PJ (1993) Differential expression and subcellular localization of protein kinase C alpha, beta, gamma, delta, and epsilon isoforms in SH-SY5Y neuroblastoma cells: modifications during differentiation. J Neurochem 60(1):289–298. 10.1111/j.1471-4159.1993.tb05850.x 8417148 10.1111/j.1471-4159.1993.tb05850.x

[43] Coleman BA, Taylor P (1996) Regulation of acetylcholinesterase expression during neuronal differentiation. J Biol Chem 271(8):4410–4416. 10.1074/jbc.271.8.4410 8626792 10.1074/jbc.271.8.4410

[44] Lawrimore CJ, Crews FT (2017) Ethanol, TLR3, and TLR4 Agonists have unique innate immune responses in Neuron-Like SH-SY5Y and Microglia-Like BV2. Alcohol Clin Exp Res 41(5):939–954. 10.1111/acer.13368 10.1111/acer.13368 PMC5407472 28273337

[45] Eleazu CO, Eleazu KC, Chukwuma S, Essien UN (2013) Review of the mechanism of cell death resulting from streptozotocin challenge in experimental animals, its practical use and potential risk to humans. J Diabetes Metab Disord 12(1):60. 10.1186/2251-6581-12-60 24364898 10.1186/2251-6581-12-60 PMC7962474

[46] Campbell A, Hamai D, Bondy SC (2001) Differential toxicity of aluminium salts in human cell lines of neural origin: implications for neurodegeneration. Neurotoxicology 22(1):63–71. 10.1016/s0161-813x(00)00007-3 11307852 10.1016/s0161-813x(00)00007-3

[47] Kawahara M, Muramoto K, Kobayashi K, Mori H, Kuroda Y (1994) Aluminium promotes the aggregation of Alzheimer’s amyloid beta-protein in vitro. Biochem Biophys Res Commun 198(2):531–535. 10.1006/bbrc.1994.1078 7507666 10.1006/bbrc.1994.1078

[48] Mustafa Rizvi SH, Parveen A, Verma AK, Ahmad I, Arshad M, Mahdi AA (2014) Aluminium induced endoplasmic reticulum stress mediated cell death in SH-SY5Y neuroblastoma cell line is independent of p53. PLoS ONE 9(5):e98409. 10.1371/journal.pone.0098409 24878590 10.1371/journal.pone.0098409 PMC4039480

[49] Piras F, Sogos V, Pollastro F, Rosa A (2024) Protective Effect of Arzanol against H2O2-Induced Oxidative Stress Damage in Differentiated and Undifferentiated SH-SY5Y Cells. Int J Mol Sci 25(13):7386. 10.3390/ijms25137386 39000492 10.3390/ijms25137386 PMC11242736

[50] Law BN, Ling AP, Koh RY, Chye SM, Wong YP (2014) Neuroprotective effects of orientin on hydrogen peroxide–induced apoptosis in SH–SY5Y cells. Mol Med Rep 9(3):947–954. 10.3892/mmr.2013.1878 24366367 10.3892/mmr.2013.1878

