# Supplementary material for: In-vitro modelling of Alzheimer’s disease using cholinergic neurons derived from human neuroblastoma (SH-SY5Y) retinoic acid-induced differentiation
Source: Mol Biol Rep. 2026 Jun 23;53(1):994. doi: 10.1007/s11033-026-12156-4 (PMC13290829; doi:10.1007/s11033-026-12156-4)
Supplement: Supplementary file 1 — Supplementary Material 1 [file 11033_2026_12156_MOESM1_ESM.docx]

Table S 1. Experimental group divisions for retinoic acid (RA)-differentiation protocol in producing cholinergic-like neurons.

| **Treatment Group** | **Media Composition** | **Differentiation Period (Days)** |
| --- | --- | --- |
| Undifferentiated Group 1 | DMEM, 1% (V/V) HI-FBS, 1% (V/V) P/S | 0 |
| Undifferentiated Group 2 | DMEM, 3% (V/V) HI-FBS, 1% (V/V) P/S | 0 |
| Differentiated Group 1 | DMEM, 1% (V/V) HI-FBS, 1% (V/V) P/S with 10μM RA | 3, 5, 7 |
| Differentiated Group 2 | DMEM, 3% (V/V) HI-FBS, 1% (V/V)P/S with 10μM RA | 3, 5, 7 |

DMEM - High Dulbecco’s Modified Eagle Medium (DMEM) (25mM D-glucose, 4.0mM L-Alanyl-L-Glutamine, 1.0mM Sodium Pyruvate). HI-FBS – Heat-inactivated foetal bovine serum. P/S – Penicillin/Streptomycin. RA – Retinoic acid.

Table S 2. Forward and reverse for RT-qPCR of cholinergic markers in RA-differentiated SH-SY5Y.

| **Target Gene** | **Primer** | **Sequence (5’-3’)** | **TM (ºC)** | **MW (g/mol)** |
| --- | --- | --- | --- | --- |
| *ChAT* | HsCHATFwd | GAGGAGCAGTTCAGGAAG | 54.3 | 6255.15 |
|  | HsCHATRev | CCAGGCGGTTGTTGAGATA | 54.9 | 5883.84 |
| *AChE* | HsACHEFwd | CCTCCTTGGACGTGTACGAT | 56.2 | 6084.00 |
|  | HsACHERev | TGATCCAGGAGACCCACAT | 55.4 | 6071.00 |
| *GAPDH* | HsGAPDHFwd | ACCACCCTGTTGCTGTAGCC | 57.7 | 6646.36 |
|  | HsGAPDHRev | GTCTCCTCTGACTTCAACAG | 61.0 | 6655.37 |

*ChAT* – Choline acetyltransferase; *AChE*- Acetylcholinesterase; *GAPDH*-Glyceraldehyde 3-phosphate dehydrogenase

Table S 3.Summary findings of compiled studies (2000-2024) on available differentiation protocol in literature.

| **No** | **Study** | **Year** | **Cell Line** | **Retinoic Acid** | **BDNF** | **Serum Percentage (%)** | **Time-Points (Days)** | **Total Days (Maximum)** | **Reference** |
| --- | --- | --- | --- | --- | --- | --- | --- | --- | --- |
| 1 | Jämsä et al., | 2004 | SH-SY5Y | 10μM | 50ng/mL | 0.0% | - | 8 | [20] |
| 2 | Encinas et al., | 2000 | SH-SY5Y | 10μM | 50ng/mL | 0.0% | 5, 10, 15, 20, 25, 30 | 30 | [21] |
| 3 | Targett et al., | 2024 | SH-SY5Y | 10μM | 50ng/mL | 1.0% | 3, 7, 10 | 10 | [8] |
| 4 | Jahn et al., | 2017 | SH-SY5Y | 50μM | 50ng/mL | N/A | - | 5 | [22] |
| 5 | Serdar et al., | 2020 | SH-SY5Y | 10μM | 50ng/mL | N/A | 5, 10 | 10 | [23] |
| 6 | Simões et al., | 2021 | SH-SY5Y | 10μM | - | 1.0% | - | 3 | [24] |
| 7 | Froster et al., | 2016 | SH-SY5Y | 10μM | 50ng/mL | 5.0% | - | 6 | [25] |
| 8 | Teppola et al., | 2015 | SH-SY5Y | 10μM | 50ng/mL | N/A | - | 10 | [12] |
| 9 | D’Aloia et al., | 2024 | SH-SY5Y | 10μM | 50 ng/mL | N/A | - | 17 | [26] |
| 10 | Pereira et al., | 2024 | SH-SY5Y | 10μM | 50ng/mL | 1.0% | - | 7 | [27] |
| 11 | Moreira et al., | 2022 | SH-SY5Y | 10μM | 50ng/mL | 1.0% | - | 7 | [28] |
| 12 | Shipley et al., | 2016 | SH-SY5Y | 10μM | 50ng/mL | 0.0% | - | 18 | [9] |
| 13 | de Medeiros et al., | 2019 | SH-SY5Y | 10μM | 50ng/mL | 1.0% | - | 7 | [15] |
| 14 | Cheung et al., | 2009 | SH-SY5Y | 10μM | - | 3.0% | - | 7 | [29] |
| 15 | Dwane et al., | 2013 | SH-SY5Y | 10μM | - | 3.0% | - | 3 | [30] |
| 16 | Dravid | 2021 | SH-SY5Y | 10μM | 50ng/mL | 3.0% | - | 10 | [31] |
| 17 | Alaylıoğlu et al., | 2024 | SH-SY5Y | 10μM | 50ng/mL | 1.0% | 4, 7 | 7 | [32] |
| 18 | Arslan et al., | 2020 | SH-SY5Y | 10μM | 50ng/mL | 2.0% | - | 11 | [33] |
| 19 | Voogd et al., | 2024 | SH-SY5Y | 10μM | 50ng/mL | 0.0% | 3, 7, 14, 21 | 21 | [34] |
| 20 | Ducray et al., | 2020 | SH-SY5Y | 10μM | - | 1.0% | - | 7 | [35] |
| 21 | Filograna et al., | 2015 | SH-SY5Y & BE (2)-M17 | 10μM | - | N/A | - | 7 | [36] |
| 22 | Hromadkova et al., | 2020 | SH-SY5Y | 10μM | - | 0.5% | - | 12 | [37] |
| 23 | Pulkrabkova et al., | 2023 | SH-SY5Y | 10μM | 50ng/mL | N/A | 1,3,6,9 | 9 | [38] |

(-) indicate that it was exclude from study. (N/A) indicate that the author did not explicitly state in the methodology.

Table S 4. Summary findings of compiled studies (2000-2024) on inclusion of mature neuronal, cholinergic, AD-related, and other neuronal markers.

| **Number** | **Study** | **Year** | **Mature Neuronal Markers** | **Check Cholinergic Marker** | **Cholinergic Markers** | **AD-Related Markers** | **Other Markers** | **Reference** |
| --- | --- | --- | --- | --- | --- | --- | --- | --- |
| 1 | Jämsä et al., | 2004 | MAPT (Tau) | No | - | MAPT (Tau), pTau Ser199, pTau Ser202, pTau Ser396, pTau Ser404, pTau Thr205, Tau-5 | GSK3B, p-GSK3B (Y216), CDK5, p35 | [20] |
| 2 | Encinas et al., | 2000 | NF-H, MAP2, MAPT (Tau) | No | - | MAPT (Tau) | – | [21] |
| 3 | Targett et al., | 2024 | TUBB3 (βIII-tubulin), SYP, DLG4 (PSD-95), MAPT (Tau) | Yes | ChAT | MAPT (Tau), pTau Ser396, AT8 | TH, SLC1A3 (GLAST), GLUL | [8] |
| 4 | Jahn et al., | 2017 | MAPT, NF-H, SYP | No | - | MAPT | NRG1, CNR1, DTNBP1, RELN, SDHA, UBC | [22] |
| 5 | Serdar et al., | 2020 | MAP2, TUBB3 (βIII-tubulin), NF-H | No | - | – | – | [23] |
| 6 | Simões et al., | 2021 | MAP2, NES (Nestin), RBFOX3 (NeuN), TUBB3 (βIII-tubulin) | No | - | – | TH, TOMM20 | [24] |
| 7 | Froster et al., | 2016 | DLG4 (PSD-95), GRIN1, RBFOX3 (NeuN), NLGN1, RET, SNAP25, SV2A, SYN1, SYP, SYT1, TUBB3 | Yes | AChE, CHRM2 | – | DDC, DBH, DRD2, EN1, KCNJ6, COMT, NES, NR4A2, PAX6, SOX2, TH, SLC6A3 (DAT) | [25] |
| 8 | Teppola et al., | 2015 | NF-68 | No | **-** | – | – | [12] |
| 9 | D’Aloia et al., | 2024 | TUBB3 (βIII-tubulin), RBFOX3 (NeuN), SYN1, SYP, CPLX1, DLG4 (PSD-95) | Yes | ChAT | – | TH | [26] |
| 10 | Pereira et al., | 2024 | TUBB3 (βIII-tubulin) | Yes | AChE | – | – | [27] |
| 11 | Moreira et al., | 2022 | TUBB3 (βIII-tubulin), MAP2 | No | - | – | – | [28] |
| 12 | Shipley et al., | 2016 | SMI-31, MAP2 | No | - | – | – | [9] |
| 13 | de Medeiros et al., | 2019 | MAPT (Tau) | Yes | SLC18A3, AChE, ChAT | PSEN1, MAPT (Tau) | CDK5, VMAT2, SLC6A3 (DAT) | [15] |
| 14 | Cheung et al., | 2009 | NSE, SYP, SAP97, RBFOX3 (NeuN), NF, MAP2 | No | **-** | – | SLC6A3 (DAT), TH | [29] |
| 15 | Dwane et al., | 2013 | TUBB3 (βIII-tubulin), GAP43 | No | **-** | – | SLC6A3 (DAT), TH | [30] |
| 16 | Dravid | 2021 | TUBB3 (βIII-tubulin), GAP43, MAPT (Tau) | No | **-** | – | – | [31] |
| 17 | Alaylıoğlu et al., | 2024 | MAPT (Tau), MAP2, SYP | No | **-** | MAPT (Tau) | TH | [32] |
| 18 | Arslan et al., | 2020 | – | No | - | – | – | [33] |
| 19 | Voogd et al., | 2024 | MAP2, SYN1/2 | No | - | – | – | [34] |
| 20 | Ducray et al., | 2020 | TUBB3 (βIII-tubulin), NF-M, MAP2 | Yes | CHT-1 | – | 5-HT, TH, NE, DBH, SNCA (α-synuclein), VMAT2 | [35] |
| 21 | Filograna et al., | 2015 | TUBB3 (βIII-tubulin), NF | No | - | – | VMAT, TH, DBH, RP11, DDC (AADC) | [36] |
| 22 | Hromadkova et al., | 2020 | MAP2, SYP, DLG4 (PSD-95), SHANK3, BSN (Bassoon) | No | - | MAPT | – | [37] |
| 23 | Pulkrabkova et al., | 2023 | Tau-5, 2G10, MAP2, TUBB3 (βIII-tubulin) | Yes | AChE | MAPT (Tau) | – | [38] |

(-) indicate that it was exclude from study. (N/A) indicate that the author did not explicitly state in the methodology. The abbreviations used are as follows: AADC/DDC, aromatic L-amino acid decarboxylase/dopa decarboxylase; AChE, acetylcholinesterase; AT8, phosphorylated tau antibody (pSer202/pThr205 tau epitope); BSN, bassoon presynaptic cytomatrix protein; CDK5, cyclin-dependent kinase 5; ChAT, choline acetyltransferase; CHT-1/CHT1, choline transporter 1; CHRM2, cholinergic receptor muscarinic 2; COMT, catechol-O-methyltransferase; CPLX1, complexin 1; DAT/SLC6A3, dopamine transporter; DBH, dopamine beta-hydroxylase; DLG4 (PSD-95/PSD95/PSD91), discs large homolog 4/postsynaptic density protein 95; DRD2, dopamine receptor D2; DTNBP1, dysbindin-1; EN1, engrailed homeobox 1; GAP43/GAP-43, growth-associated protein 43; GLAST/SLC1A3, glutamate aspartate transporter; GLUL, glutamate-ammonia ligase (glutamine synthetase); GRIN1, glutamate ionotropic receptor NMDA type subunit 1; GSK3B, glycogen synthase kinase 3 beta; GSK3B Y216, phosphorylated glycogen synthase kinase 3 beta (Tyr216); KCNJ6, potassium inwardly rectifying channel subfamily J member 6; MAP2/MAP-2, microtubule-associated protein 2; MAPT (Tau), microtubule-associated protein tau; NES/NESTIN, nestin intermediate filament protein; NF, neurofilament; NF-H, neurofilament heavy chain; NF-M, neurofilament medium chain; NF-68, neurofilament 68 kDa; NeuN/RBFOX3, RNA binding fox-1 homolog 3/neuronal nuclei marker; NLGN1, neuroligin 1; NR4A2, nuclear receptor subfamily 4 group A member 2; NSE, neuron-specific enolase; P35, cyclin-dependent kinase 5 regulatory subunit-associated protein 1; PAX6, paired box 6; PSEN1, presenilin 1; pS396, phosphorylated tau Ser396; RELN, reelin; RET, RET proto-oncogene receptor tyrosine kinase; RP11, RP11 long non-coding RNA region; SAP97, synapse-associated protein 97; SDHA, succinate dehydrogenase complex flavoprotein subunit A; SHANK3, SH3 and multiple ankyrin repeat domains protein 3; SLC18A3, vesicular acetylcholine transporter; SMI-31, phosphorylated neurofilament marker antibody; SNAP25, synaptosomal-associated protein 25; SNCA (α-synuclein), alpha-synuclein; SOX2, SRY-box transcription factor 2; SV2A, synaptic vesicle glycoprotein 2A; SYN1, synapsin I; SYP, synaptophysin; SYT1, synaptotagmin I; TH, tyrosine hydroxylase; TOMM20, translocase of outer mitochondrial membrane 20; TUBB3 (βIII-tubulin), tubulin beta-3 chain; UBC, ubiquitin C; VMAT/VMAT2, vesicular monoamine transporter 2; Tau-5, total tau antibody clone Tau-5; and 2G10, tau antibody clone 2G10; NE, noradrenaline; 5-HT, serotonin.
